# ANGPTL2 knockdown induces autophagy to relieve alveolar macrophage pyroptosis by reducing LILRB2‐mediated inhibition of TREM2

**DOI:** 10.1111/jcmm.18280

**Published:** 2024-05-17

**Authors:** Fan Yang, Muhu Chen, Ying Liu, Yingchun Hu, Yangxi Chen, Youwei Yu, Lu Deng

**Affiliations:** ^1^ Department of Emergency Medicine The Affiliated Hospital of Southwest Medical University Luzhou China; ^2^ Department of Thyroid Surgery The Affiliated Hospital of Southwest Medical University Luzhou China

**Keywords:** ANGPTL2, autophagy, LILRB2, lung injury, pyroptosis, TREM2

## Abstract

Acute lung injury (ALI) is featured with a robust inflammatory response. Angiopoietin‐like protein 2 (ANGPTL2), a pro‐inflammatory protein, is complicated with various disorders. However, the role of ANGPTL2 in ALI remains to be further explored. The mice and MH‐S cells were administrated with lipopolysaccharide (LPS) to evoke the lung injury in vivo and in vitro. The role and mechanism of ANGPTL was investigated by haematoxylin–eosin, measurement of wet/dry ratio, cell count, terminal deoxynucleotidyl transferase deoxyuridine triphosphate (dUTP) nick end labeling, reverse transcription quantitative polymerase chain reaction, immunofluorescence, enzyme‐linked immunosorbent assay, detection of autophagic flux and western blot assays. The level of ANGPTL2 was upregulated in lung injury. Knockout of ANGPTL2 alleviated LPS‐induced pathological symptoms, reduced pulmonary wet/dry weight ratio, the numbers of total cells and neutrophils in BALF, apoptosis rate and the release of pro‐inflammatory mediators, and modulated polarization of alveolar macrophages in mice. Knockdown of ANGPTL2 downregulated the level of pyroptosis indicators, and elevated the level of autophagy in LPS‐induced MH‐S cells. Besides, downregulation of ANGPTL2 reversed the LPS‐induced the expression of leukocyte immunoglobulin (Ig)‐like receptor B2 (LILRB2) and triggering receptor expressed on myeloid cells 2 (TREM2), which was reversed by the overexpression of LILRB2. Importantly, knockdown of TREM2 reversed the levels of autophagy‐ and pyroptosis‐involved proteins, and the contents of pro‐inflammatory factors in LPS‐induced MH‐S cells transfected with si ANGPTL2, which was further inverted with the treatment of rapamycin. Therefore, ANGPTL2 silencing enhanced autophagy to alleviate alveolar macrophage pyroptosis via reducing LILRB2‐mediated inhibition of TREM2.

## 1 INTRODUCTION

Acute lung injury (ALI) is a grievous respiratory disorder, which can be resulted from various direct or indirect cues, such as infection, trauma, shock and noxious gas inhalation.^1^ The clinical symptoms of ALI include shortness of breath, stubborn hypoxemia and alveolar and parenchymal edema.^2^ If ALI is not controlled, it is possibly to advance to acute respiratory distress syndrome (ARDS). It is reported that about 10% of patients admitted to the intensive care unit (ICU) are diagnosed with ARDS, thus, ALI/ARDS is the primary cause of death in critical ill patients.^3^ Several agents have been applied for the treatment of ALI, such as dexamethasone, prednisone and prednisolone, however, a variety of adverse effects are indicated, including gastric ulcers, coagulation diseases and osteoporosis.^4^, ^5^ Besides, mechanical ventilation is another choice for the patients with ALI/ARDS.^3^, ^6^ Nevertheless, the mortality rate of ALI/ARDS is as high as 35%–46%.^3^ Hence, it is pressing to clarify the underlying mechanisms and develop available therapies for ALI/ARDS.

ALI/ARDS is characterized by uncontrolled and excess inflammatory lung injury, in which inflammation is presented in the whole range of pathological stages of ALI/ARDS.^7^ Inflammation cause destruction of lung tissue, neutrophil infiltration, and release of pro‐inflammatory mediators and the relative abundance of anti‐ and pro‐inflammatory factors depends on the severity and prognosis of lung injury.^8^, ^9^ As the primary immune cells in the airways, alveolar macrophages maintain a homeostatic condition.^10^ Alveolar macrophages start innate immune responses and activate adaptive immunity to generate generous chemokines and inflammatory factors that initiate and expand the inflammatory responses in the lung.^11^ Thus, strategies targeting inflammation are significant approaches for the treatment of ALI/ARDS.

Angiopoietin‐like protein 2 (ANGPTL2) is a glycoprotein, which is abundantly expressed in lung, adipose tissue, heart and skeletal muscle.^12^ ANGPTL2 is identified as a pro‐inflammatory protein that is strongly involved in various diseases. Activation of ANGPTL2 evokes inflammation of the vasculature,^13^ and deletion of ANGPTL2 relieves adipose tissue inflammation in obese mice.^14^ ANGPTL2 is demonstrated to be related to chronic inflammation in dermatomyositis^15^ and synovial inflammation in rheumatoid arthritis.^16^ Also, ANGPTL2 mediates atherosclerosis by promoting the progression of chronic endothelial/vascular inflammation.^17^ Thus, circulating levels of ANGPTL2 are notably increased in a variety of inflammatory diseases and have been identified as a clinical marker beyond systemic inflammation.^18^ Moreover, deficiency of ANGPTL2 dampens paraquat‐elicited lung injury through regulating oxidative stress, inflammation and fibrosis via NF‐κB pathway,^19^ indicating that ANGPTL2 is a potential therapeutical target of ALI. It is demonstrated that ANGPTL2 can bind and activate leukocyte immunoglobulin (Ig)‐like receptor B2 (LILRB2).^20^ LILRB2 has three inhibitory motifs based on the immune receptor tyrosine (ITIM) and is found to be selectively expressed in monocytes, macrophages, and dendritic cells.^20^ ANGPTL2 has been revealed that evokes the expression of inflammatory mediators in synovial cells through LILRB2, thereby inducing synovial inflammation.^21^ In addition, LILRB2 is reported that negatively regulates the triggering receptor expressed on myeloid cells 2 (TREM2) signalling in microglia.^22^ TREM2 is a widely expressed pattern‐recognition receptor on macrophages, dendritic cells, and microglia and is considered an important therapeutic target for neurodegenerative diseases, infectious disorders, and tumour immunotherapy.^23^ Based on the findings, we speculated that ANGPTL2/LILRB2/TREM2 axis might regulate ALI.

Thus, in the present study, mice and MH‐S cells were stimulated with lipopolysaccharide (LPS) to evoke the lung injury in vivo and in vitro. The role and mechanism of ANGPTL2 in ALI were probed at the histological, cytopathological and molecular level.

## 2 MATERIALS AND METHODS

### 2.1 Animals

Healthy C57BL/6 mice (8‐weeks‐old, 20 ± 2 g) were provided from Cyagen (Jiangsu, China) and fed in a temperature‐controlled laboratory conditions with the 12‐h cycle of light–dark and freely fed with rodent chow. Age‐ and sex‐matched mice were randomly assigned into the experimental groups. ANGPTL2^−/−^ mice (ANGPTL2 KO mice) were generated via the CRISPR‐Cas9 method. Briefly, C57BL/6 mice were administrated with recombinant Cas9 and guide RNAs targeting ANGPTL2 exon1 by the pronuclear microinjection. The offspring of ANGPTL2 KO mice were identified by the genotype and the sequence, and the identified offspring were used for the following study. All animal experiments were in accord with the Guide for the Care and Use of Laboratory Animals^24^ and authorized by the Animal Research Ethics Committee of the Affiliated Hospital of Southwest Medical University.

### 2.2 Animal group and treatment

Mice were anaesthetised with isoflurane (catalogue number: R510‐22, RWD, Guangdong, China) and then subjected to the orotracheal intubation with a 20‐gauge intravenous catheter. LPS (0111:B4, catalogue number: L4391, Sigma‐Aldrich, St. Louis, MO, USA) with a dose of 5 mg/kg was instilled into the lung of mice, while the same dose of phosphate buffered saline (PBS, catalogue number: P1020, Solarbio, Beijing, China) was acted as the control. Mice were randomly divided into four groups, including control, control+ANGPTL2 KO, LPS and LPS + ANGPTL2 KO with eight mice in each group. C57BL/6 mice injected with no‐targeted sgRNA were used in control and LPS groups, and then treated with PBS and LPS, respectively. C57BL/6 mice administrated with recombinant Cas9 and guide RNAs targeting ANGPTL2 exon1 used in control+ANGPTL2 KO and LPS + ANGPTL2 KO groups, and then subjected with PBS and LPS, respectively. To achieve the complete distribution of LPS or PBS throughout the lungs, mice were ventilated mechanically with room air using a rodent ventilator (VentElite, Harvard Apparatus, MA, USA) according to the previous report.^25^ The volume‐controlled setting parameters were as following: 120 breaths/min, 7 mL/kg body weight, and a positive end‐expiratory pressure (PEEP) of 2 cm H_2_O for 10 min. After 6 h based on the previous study suggested that the peak of the inflammatory lung response occurs around 6 hours after LPS administration,^26^ mice were weighted using an electronic scale, and then sacrificed by inhaling the excess isoflurane. The bronchoalveolar lavage fluid (BALF) by flushing with 1 mL PBS and lung tissues of mice were harvested for the following assays.

### 2.3 Pathological staining

The right lung lobes were removed and immersed into 4% paraformaldehyde (catalogue number: P1110, Solarbio) for 2 days, and then treated with the dehydration and embeddedness. The paraffin‐embedded tissues sliced into 5 μm sections, which were stained by haematoxylin–eosin (HE) (catalogue number: G1120, Solarbio). After being mounted with neutral resin (catalogue number: G8590, Solarbio), slices were imaged by a digital trinocular camera microscope (CX23, Olympus, Tokyo, Japan). The pathological outcomes were assessed based on four indexes (haemorrhage, thickening of the alveolar septa, alveolar oedema and leukocyte infiltration) as described previously.^27^ Each index was scored ranging from 0 to 3 based on severity, in which 0–3 indicated normal, mild, moderate and severe respectively. The severity of lung injury was assessed by the sum of each score.

### 2.4 Measurement of wet/dry (W/D) ratio of lung tissue

The lower lobe of the left lung was isolated, and immediately weighed by an electronic balance. Subsequently, the lung tissues were dried at 58°C. After 48 h, the lung tissues were reweighed for the quantification of the wet‐to‐dry ratio with the formula of wet weight/dry weight.^28^

### 2.5 Count of the cell numbers

The precipitated cells in BALF were yielded through the centrifugation at 400 × g for the measurement of the numbers of total cells and neutrophils in BALF. Precipitated cells were resuspended in PBS and sowed on 15‐mm glass slides. After cells were maintained for 1 h at 37°C to stick to the slides, Wright's‐Giemsa staining (catalogue number: G1020, Solarbio) was performed to count the numbers of total cells and neutrophils.

### 2.6 Terminal deoxynucleotidyl transferase deoxyuridine triphosphate (dUTP) nick end labeling (TUNEL) assays

The paraffin slices were dewaxed with xylene and dehydrated with graded ethanol, and then hatched with proteinase K (catalogue number: P9460, Solarbio) for half an hour at room temperature. After being rinsed with PBS for three times, the slices were administrated with 2% H_2_O_2_ min at room temperature for 20. Next, the slices were rinsed with PBS thrice, and hatched with TUNEL reaction mixtures (catalogue number: C1090, Beyotime, Shanghai, China) at 37°C for 1 h. Subsequently, the slices were hatched with 50 μL Streptavidin‐HRP (catalogue number: A0305, Beyotime) at room temperature for 30 min, and covered with 500 μL DAB (catalogue number: P0202, Beyotime) at room temperature for 30 min. The slices were re‐stained with Mounting Medium, antifading (with DAPI) (catalogue number: S2110, Solarbio) and imaged by a fluorescence microscope (IX71, Olympus). The percent of apoptosis cells was assessed via TUNEL positive cells/the total cells number.

### 2.7 Reverse transcription quantitative polymerase chain reaction (RT‐qPCR)

According to the previous report,^29^ total RNA from BALF was prepared by TRIzol reagent (catalogue number: 15596026, Thermo Fisher Scientific, Waltham, MA, USA), and then reversely transcribed into cDNA with the Bio‐Rad ScripTM cDNA Synthesis Kit (catalogue number: 1708890, Bio‐Rad Laboratories, Inc., Hercules, CA, USA) based on the operation instruction. RT‐qPCR assay was conducted on the Bio‐Rad CFX Manager software (Bio‐Rad Laboratories, Inc.) with 2 × SYBR Master mix (catalogue number: RR820A, Takara, Dalian, China). The levels of tumour necrosis factor (TNF)‐α, interleukin (IL)‐6, il‐18 and monocyte chemotactic protein‐1 (MCP‐1) were analysed by the 2^−ΔΔCT^ method after being normalized with the *GAPDH*. The sequences of primer were shown in Table 1.

**TABLE 1:** The primer sequences of genes used in the present study.

Name	Forward (5′‐3′)	Reverse (5′‐3′)
TNF‐α	GCGGTGCCTATGTCTCAGCCTCTTCT	GGTGGTTTGTGAGTGTGAGGGTCTGG
IL‐1β	TCTCGCAGCACATCAACAAGAGC	GGAAGGTCCAAGGGAAAGACACAGGT
IL‐6	TGGAGCCCACCAAGAACGATAGTCAA	TGTCACCAGCATCAGTCCCAAGAAGG
MCP‐1	CCTGCTGCTACTCATTCACCA	CAGACCTCTCTCTTGAGCTTGG
GAPDH	ATGGTGAAGGTCGGTGTGAACGGATT	GTCTCGCTCCTGGAAGATGGTGATGG

### 2.8 Immunofluorescence (IF) assay

Mice were perfused with pre‐cold 0.1 M PBS transcardially and immediately perfused with pe‐cold 4% paraformaldehyde. Then, lung tissues were excised and immobilized in 4% paraformaldehyde overnight. Next, lung tissues were embedded into OCT (catalogue number: 4583, Sakura, CA, USA) and sectioned into 5 μm slices. Slices were treated with BSA blocking buffer (catalogue number: SW3015, Solarbio) and 0.2% Triton X‐100 (catalogue number: T8200, Solarbio), as well as the primary antibodies (including Alexa Fluor® 488 Rat monoclonal [FA‐11] to CD68 (1:250, catalogue number: ab201844, Abcam, Cambridge, UK) and Rabbit polyclonal to Mannose Receptor (CD206) with a concentration of 1 μg/mL (catalogue number: ab64693, Abcam)) overnight at 4°C. Slices were hatched with Goat Anti‐Rabbit IgG H&L (Alexa Fluor® 647) (1:500, catalogue number: ab150079, Abcam) at room temperature for 1 h after washed with PBS thrice. Slices were stained with Mounting Medium, antifading (with DAPI) (catalogue number: S2110, Solarbio) and imaged by a fluorescence microscopy (IX71, Olympus).

### 2.9 Cell treatment

Mouse alveolar macrophages MH‐S cells were acquired from Procell (catalogue number: CL‐0597, Wuhan, China), and grown in MH‐S cell specific media (catalogue number: CM‐0597, Procell) in an incubator at 37°C with 5% carbon dioxide (CO_2_). MH‐S cells were administrated with 10 μg/mL LPS for 4 h to evoke lung injury in vitro based on the previous report.^30^ Small interfering ribonucleic acid (siRNA) targeting ANGPTL2 (si ANGPTL2) and TREM2 (si TREM2), as well as the corresponding negative control (si NC) were prepared by GenePharma (Shanghai, China). The sequences of LILRB2 were sub‐cloned into pcDNA vector plasmids for the overexpression of LILRB2. The transfection was executed on MH‐S cells using Lipofectamine 3000 (catalogue number: L3000075, Invitrogen, Carlsbad, CA, USA) based on the previous description.^31^ Cells were harvested for the subsequent assays after 48 h of transfection. Besides, to explore the role of autophagy, MH‐S cells were incubated with rapamycin (an autophagy activator) (catalogue number: IR0010, Solarbio) with a concentration of 3 μM for 12 h based on the previous study.^32^

### 2.10 Enzyme‐linked immunosorbent assay (ELISA)

The concentration of IL‐1β in cell culture supernatant was detected with Mouse IL‐1β ELISA Kit (catalogue number: PI301, Beyotime), while the concentration of TNF‐α, IL‐6, IL‐18 and MCP‐1 in both BALF and cell culture supernatant were examined by Mouse TNF‐α ELISA Kit (catalogue number: PT512, Beyotime), Mouse IL‐6 ELISA Kit (catalogue number: PI326, Beyotime), Mouse IL‐18 ELISA Kit (catalogue number: PI553, Beyotime) and Mouse CCL2/JE/MCP‐1 ELISA Kit (catalogue number: PC125, Beyotime) in keeping with the working instructions. The absorbance was determined with a microplate reader (Thermo Fisher Scientific) at 450 nm.

### 2.11 Detection of autophagic flux

MH‐S cells were inoculated in EZ Slide 8 Well Glass (catalogue number: PEZGS0816, Merk, German) at a density of 10^4^/mL, and maintained with 5% CO_2_ at 37°C for 24 h. 0.1 μL mRFP‐GFP‐LC3 adenovirus (Hanbio Technology, Shanghai, China) with a multiplicity of infection (MOI) of 100 was used to transfect MH‐S cells. 1/2 volume of fresh culture media were added when virus infection, and then another 1/2 volume of fresh culture media were appended after 4 h of lentivirus infection. After 24 h of lentivirus infection, fresh culture media were changed. The fluorescence signals in cells were determined by a fluorescence microscopy (IX71, Olympus) after 48 h of lentivirus infection.

### 2.12 Western blot

Based on the previous studies,^33^, ^34^ lung tissues and MH‐S cells were administrated with RIPA lysis buffer (catalogue number: R0010, Solarbio) to yield the total proteins, whose concentrations were determined with BCA Protein Assay Kit (catalogue number: PC0020, Solarbio). Protein samples (20 μg) were dissolved with 10% sodium dodecyl sulfate‐polyacrylamide gel electrophoresis (SDS‐PAGE) and then shifted onto PVDF membranes (catalogue number: IPVH00010, EMD Millipore, Billerica, MA, USA). The membranes were sealed in 5% BSA Blocking Buffer (catalogue number: SW3015, Solarbio) at room temperature for half an hour, and then treated with primary antibodies overnight at 4°C. Subsequently, the membranes were administrated with the secondary antibodies Goat Anti‐Rabbit IgG H&L (HRP) (1:20000, catalogue number: ab6721, Abcam, Cambridge, UK) at room temperature for 1 h. GAPDH served as the internal reference. The bands were determined by a BeyoECL Plus kit (catalogue number: P0018S, Beyotime), and the grey value was quantified with Image‐ProPlus software (Media Cybernetics, Inc., Rockville, MD, USA). The primary antibodies contained rabbit polyclonal to ANGPTL2 (1:1000, ab199133, Abcam), rabbit monoclonal to NLRP3 (1:1000, ab263899, Abcam), rabbit monoclonal to caspase‐1 (1:1000, 3866, Cell Signalling Technology, Inc., Danvers, MA, USA), rabbit monoclonal to cleaved Gasdermin D (1:1000, 10,137, Cell Signalling Technology), rabbit polyclonal to cytochrome c (1:1000, ab90529, Abcam), rabbit polyclonal to LC3I/II (1:1000, 4108), rabbit polyclonal to LILRB2 (1:2000, PA5‐116507, Thermo Fisher Scientific), rabbit monoclonal to TREM2 (1:1000, ab305103, Abcam) and rabbit monoclonal to GAPDH (1:10000, ab181602, Abcam).

### 2.13 Statistical analysis

SPSS 20.0 software (IBM Corp., Armonk, NY, USA) was employed for the statistical analysis. Data were presented as mean ± standard deviation (SD), and analysed by the unpaired Student's *t*‐test (for two groups) or one‐way analysis of variance (ANOVA) with Dunnett's post hoc test (for three or more groups) followed by post hoc Bonferroni test. *p* < 0.05 was defined as the significant differences.

## 3 RESULTS

### 3.1 Knockout of ANGPTL2 alleviates lung injury in mice

**FIGURE 1:**
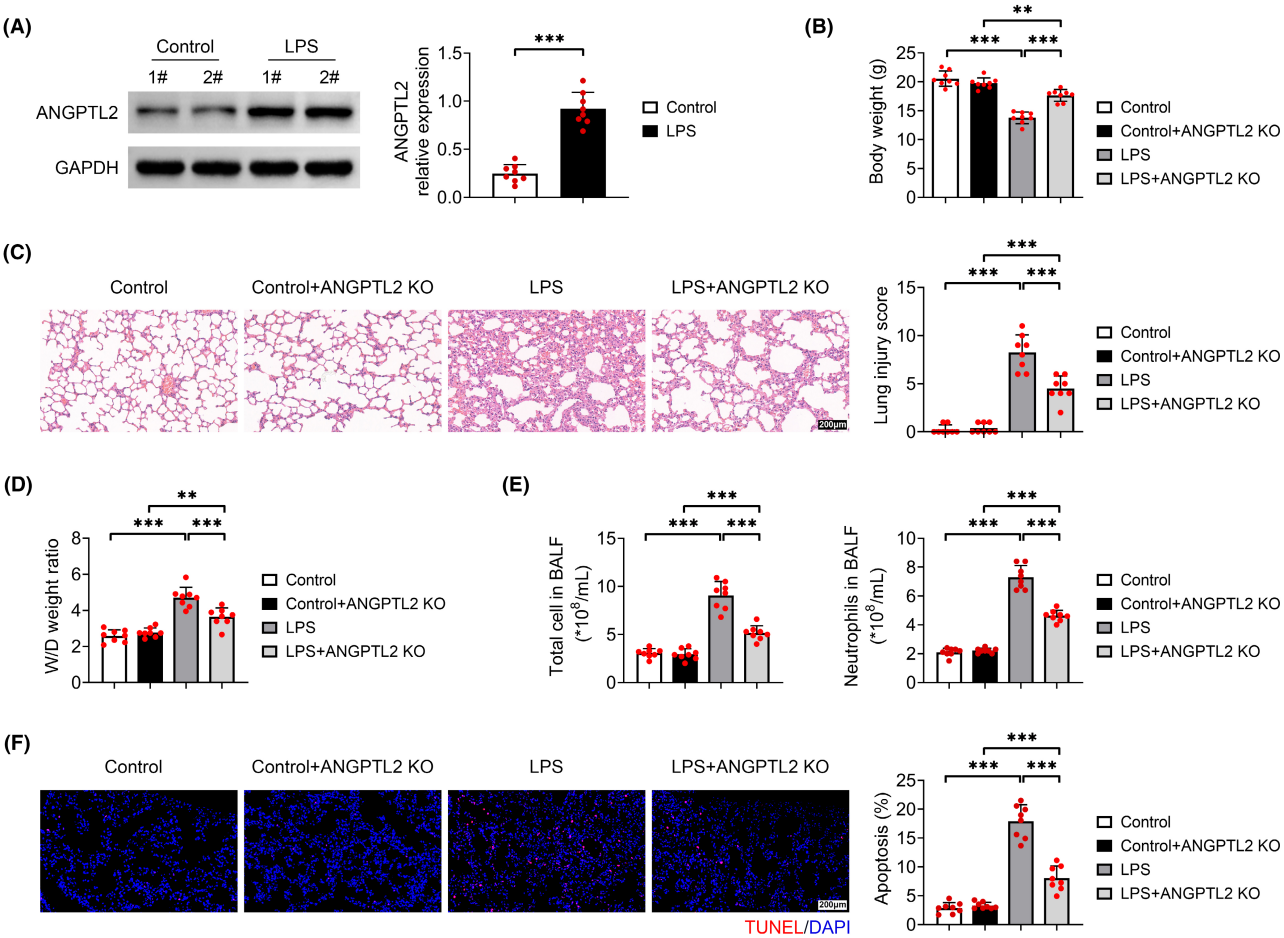
Knockout of ANGPTL2 relieved lung injury in mice. Mice were injected with LPS to induce a lung injury model, and ANGPTL2 KO mice were generated through the CRISPR‐Cas9 method. (A) The relative protein expression of ANGPTL2 was examined by western blot. Results were expressed after normalized with GAPDH. (B) Measurement of body weight. (C) The pathological manifestations of lung tissues were assessed by HE staining, and scored. Scale bar = 200 μm. (D) The pulmonary wet/dry weight ratio was measured to evaluate the pulmonary edema. (E) The numbers of total cells and neutrophils in BALF were counted by Wright's‐Giemsa staining. (F) The apoptosis rate of lung tissues was determined by TUNEL assays. Scale bar = 200 μm. Data were shown as averages ± SD. **p < 0.01 and ***p < 0.001.

After mice were stimulated with LPS to evoke a lung injury model, the relative protein level of ANGPTL2 was notably increased in lung tissues relative to that in mice injected with PBS (*p* < 0.001) (Figure 1A), indicating that ANGPTL2 might serve a role in the lung injury. To investigate the role of ANGPTL2 in the lung injury, ANGPTL2 KO mice were generated through the CRISPR‐Cas9 method. Body weight of LPS‐induced mice was prominently declined as compared with that of control mice (*p* < 0.001), which was significantly rescued with the deletion of ANGPTL2 (*p* < 0.001) (Figure 1B). Compared with the control mice, pathological manifestations, such as alveolar septal thickening and haemorrhage were observed in the lung tissues in the LPS‐induced mice, which were obviously relieved with the knockout of ANGPTL2 (Figure 1C). Correspondingly, ANGPTL2 deficiency prominently decreased the LPS‐induced lung injury score (*p* < 0.001) (Figure 1C). The pulmonary W/D weight ratio was markedly enhanced in LPS‐elicited mice relative to that in mice injected with PBS, which was notably reduced with the ablation of ANGPTL2 (*p* < 0.001) (Figure 1D). Similar outcomes were discovered in the numbers of total cells and neutrophils in BALF (*p* < 0.001) (Figure 1E). Besides, depletion of ANGPTL2 significantly declined the LPS‐induced apoptosis rate of lung tissues (*p* < 0.001) (Figure 1F). All these above‐mentioned indicators had no statistical difference between the control mice and control+ANGPTL2 KO mice, while a prominent induction in these indicators was observed from control+ANGPTL2 KO mice to LPS + ANGPTL2 KO mice (*p* < 0.01) (Figure 1B–F). Thus, knockout of ANGPTL2 mitigated lung injury in mice.

### 3.2 Knockout of ANGPTL2 relieves the storm of inflammatory factors in the lungs

**FIGURE 2:**
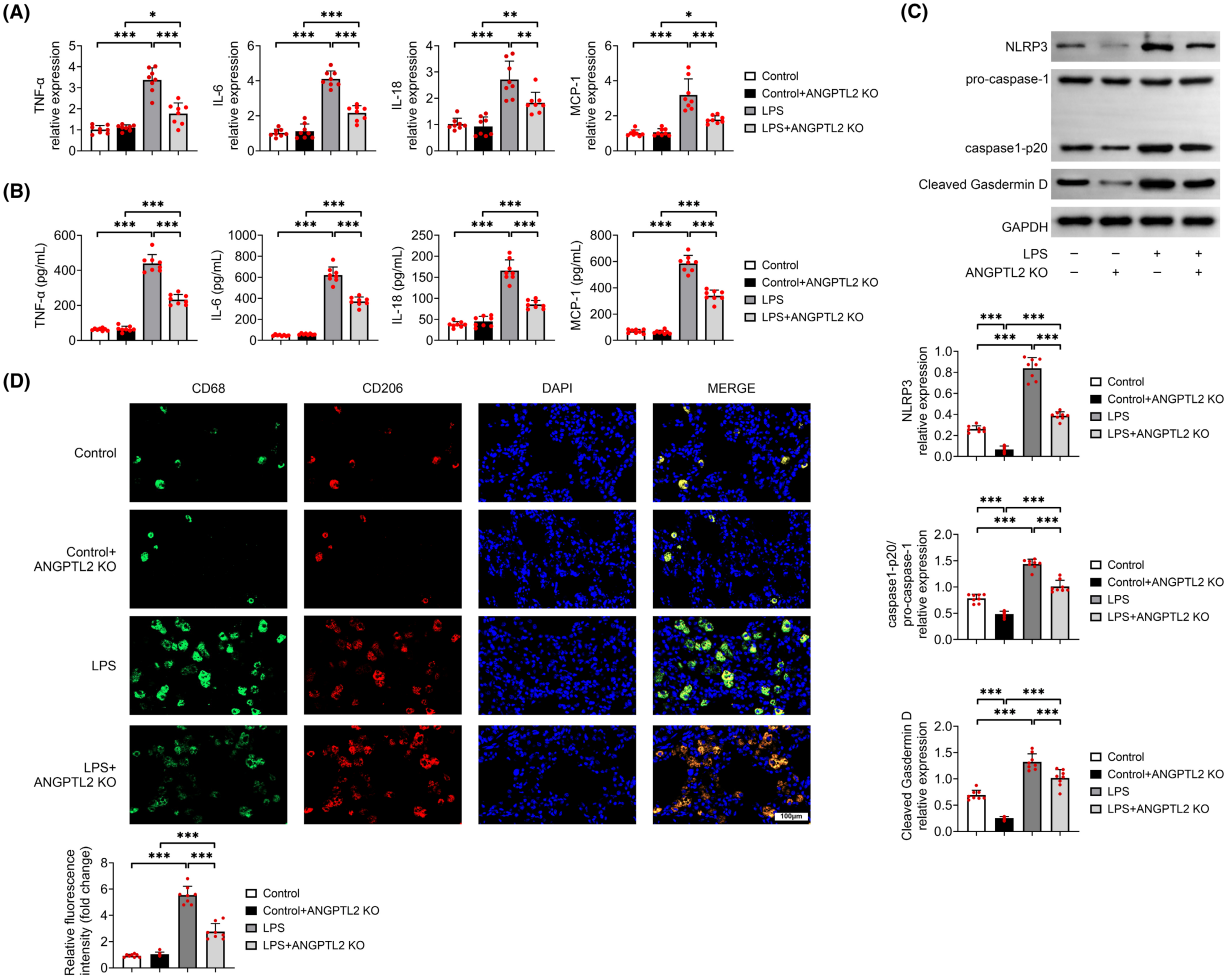
ANGPTL2 deficiency restrained the releases of pro‐inflammatory cytokines from lung tissue via modulating the polarization of alveolar macrophages. (A) The relative mRNA expressions of *TNF‐α*, *IL‐6*, *IL‐18* and *MCP‐1* were examined by RT‐qPCR. Results were expressed after normalized with *GAPDH*. (B) The concentrations of TNF‐α, IL‐6, IL‐18 and MCP‐1 in BALF were measured by ELISA. (C) The relative protein expression of NLRP3, pro‐caspase‐1, caspase1‐p20 and cleaved Gasdermin D was detected by western blot. Results were expressed after normalized with GAPDH. (D) The expressions of the biomarkers of M1 macrophages (CD68) and M2 macrophages (CD206) in lung tissues were determined by immunofluorescence assays. Cell nucleus were stained by DAPI (blue), CD68 was labelled by the green fluorescence, and CD206 was labelled by the red fluorescence. Scale bar = 100 μm. Data were shown as averages ± SD. *p < 0.05, **p < 0.01 and ***p < 0.001.

Inflammation is a significant factor during lung injury. To survey the action of ANGPTL2 in inflammation in lung injury, the contents of pro‐inflammatory mediators, containing TNF‐α, IL‐6, IL‐18 and MCP‐1 in BALF were measured. The relative mRNA expressions of TNF‐α, IL‐6, IL‐18 and MCP‐1 were markedly increased in BALF from LPS‐induced mice relative to that from control mice (*p* < 0.001), which were notably counteracted with the ANGPTL2 deficiency (*p* < 0.01) (Figure 2A). Analogical results were also indicated in the contents of TNF‐α, IL‐6, IL‐18 and MCP‐1 in BALF (Figure 2B). Besides, ablation of ANGPTL2 neutralized the expression level of NLRP3, caspase1‐p20/pro‐caspase‐1 and cleaved Gasdermin D in the LPS‐induced mice (*p* < 0.001) (Figure 2C). Since alveolar macrophages are the main immune cells in the airway, the polarization of macrophages was determined in lung tissue. As shown in Figure 2D, substantial infiltration of M1 macrophages (CD68) and M2 macrophages (CD206) were discovered in lung tissue after mice were challenged with LPS, while the ablation of ANGPTL2 visibly decreased the M1 macrophages infiltration into lung tissue. However, the fluorescence intensity of CD206 was even slightly enhanced in LPS + ANGPTL2 KO group relative to that in LPS group (Figure 2D). In addition, all these above‐mentioned indicators in control+ANGPTL2 KO mice were notably promoted with the treatment of LPS (*p* < 0.05) (Figure 2A–D). The expression level of NLRP3, caspase1‐p20/pro‐caspase‐1 and cleaved Gasdermin D was markedly reduced in control mice with the knockout of ANGPTL2 (*p* < 0.001), while there was no statistical difference in the level of TNF‐α, IL‐6, IL‐18 and MCP‐1, as well as the fluorescence intensity between the control mice and control+ANGPTL2 KO mice (Figure 2A–D). Therefore, these results indicated that knockout of ANGPTL2 modulated polarization of alveolar macrophages to suppress the pro‐inflammatory cytokines release.

### 3.3 Downregulation of ANGPTL2 mitigates LPS‐induced pyroptosis in MH‐S cells

**FIGURE 3:**
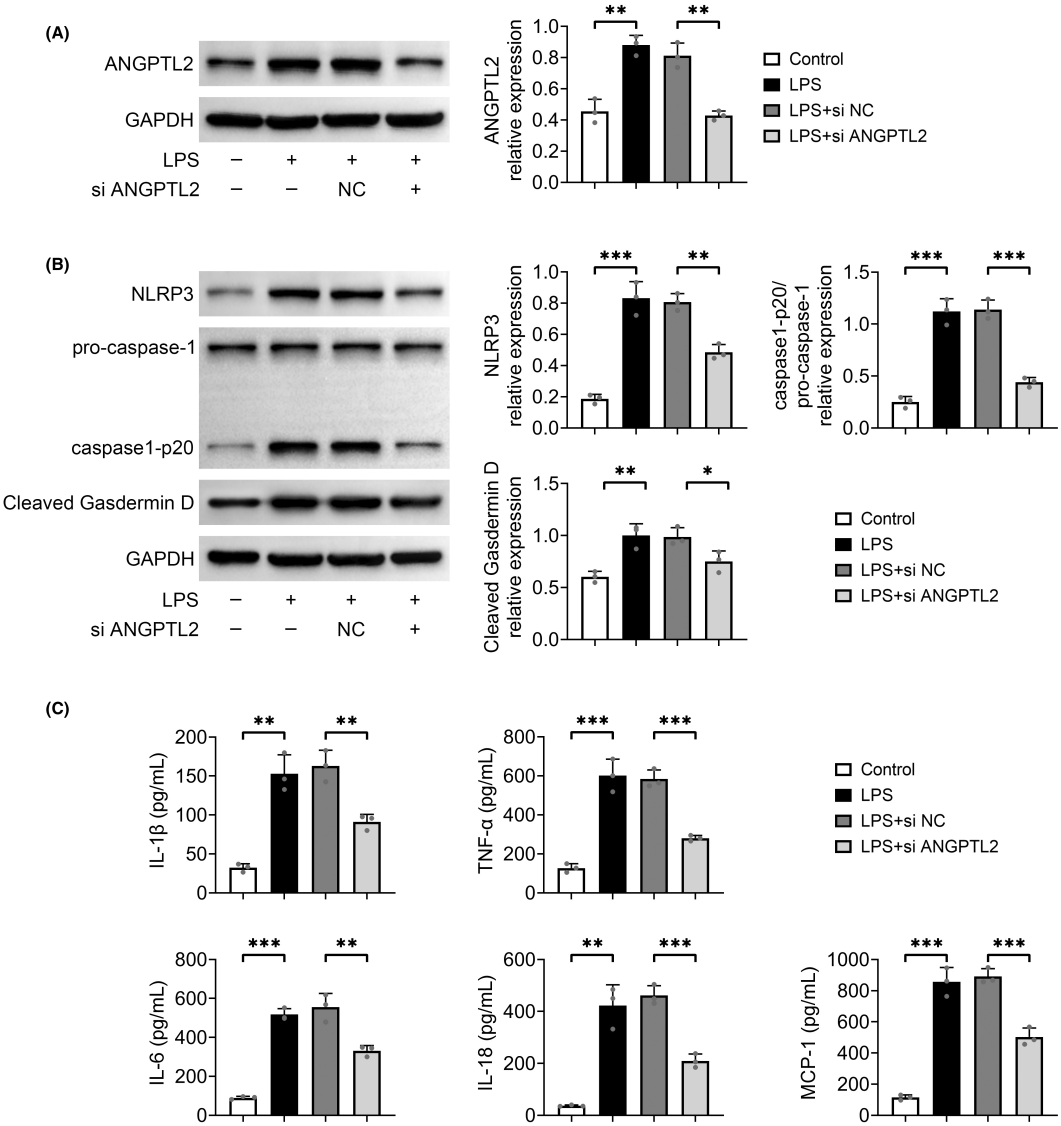
Silencing of ANGPTL2 reduced LPS‐induced pyroptosis in MH‐S cells. MH‐S cells were administrated with 10 μg/mL LPS for 4 h to induce lung injury in vitro, and si ANGPTL2 was transfected into MH‐S cells by using Lipofectamine 3000 to downregulate the level of ANGPTL2. Cells were harvested after 48 h of transfection for the analysis. (A) The relative protein expression of ANGPTL2 in MH‐S cells was examined by western blot. Results were expressed after normalized with GAPDH. (B) The relative protein expression of NLRP3, pro‐caspase‐1, caspase1‐p20 and cleaved Gasdermin D in MH‐S cells was determined by western blot. Results were expressed after normalized with GAPDH. (C) The contents of IL‐1β, TNF‐α, IL‐6, IL‐18 and MCP‐1 in cellular supernatant of MH‐S cells were measured by ELISA. Data were shown as averages ± SD. *p < 0.05, **p < 0.01 and ***p < 0.001.

To deeply survey the mechanism of ANGPTL2 in lung injury, mouse alveolar macrophages MH‐S cells were hatched with LPS to evoke a lung injury in vitro. LPS management leaded to a prominent increase in the relative protein level of ANGPTL2 in MH‐S cells (*p* < 0.01), which was markedly neutralized with the knockdown of ANGPTL2 (*p* < 0.01) (Figure 3A). It is demonstrated that macrophages may be subjected to pyroptosis accompanied by an intense inflammatory response. Thus, the expressions of the biomarkers of pyroptosis, including NLRP3, pro‐caspase‐1, caspase1‐p20 and cleaved Gasdermin D were examined through western blot. A remarkable enhancement in the relative protein level of NLRP3, pro‐caspase‐1/caspase1‐p20 and cleaved Gasdermin D was found in the LPS‐treated MH‐S cells (*p* < 0.01), which was prominently offset by the downregulation of ANGPTL2 (*p* < 0.05) (Figure 3B). Moreover, silencing of ANGPTL2 significantly reduced the LPS‐induced the contents of pro‐inflammatory mediators (IL‐1β, TNF‐α, IL‐6, IL‐18 and MCP‐1) in cellular supernatant of MH‐S cells (*p* < 0.01) (Figure 3C). Hence, downregulation of ANGPTL2 declined LPS‐evoked pyroptosis in MH‐S cells.

### 3.4 Silencing of ANGPTL2 upregulates autophagy in LPS‐elicited MH‐S cells

**FIGURE 4:**
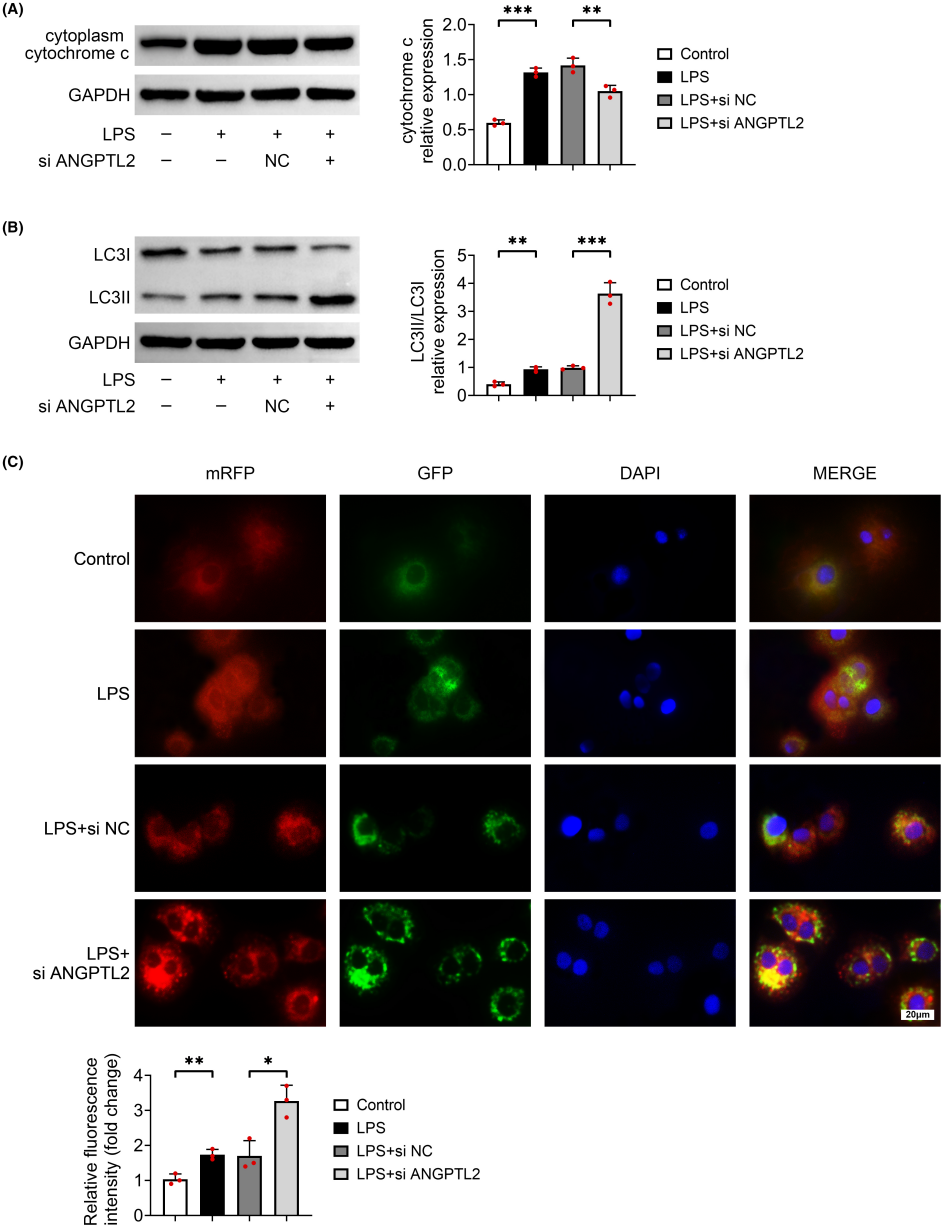
Downregulation of ANGPTL2 enhanced autophagy in LPS‐induced MH‐S cells. (A) The relative protein expression of cytoplasm cytochrome c was determined by western blot. Results were expressed after normalized with GAPDH. (B) The relative protein level of LC3II and LC3I was examined by western blot. Results were expressed after normalized with GAPDH. (C) The autophagic flux was investigated by transfecting mRFP‐GFP‐LC3 adenovirus into MH‐S cells. Autophagosomes were labelled with yellow puncta and autolysosomes were appeared as red puncta. Scale bar = 20 μm. Data were shown as averages ± SD. *p < 0.05, **p < 0.01 and ***p < 0.001.

Given that NLRP3 plays a crucial role in autophagy, the indicators related in autophagy were detected in MH‐S cells. Results from Figure 4A revealed that the relative protein level of cytoplasm cytochrome c was markedly elevated in LPS‐induced MH‐S cells (*p* < 0.001), which was observably counteracted with the knockdown of ANGPTL2 (*p* < 0.01). Besides, LPS treatment evoked an arresting elevation in the relative protein level of LC3II/LC3I (*p* < 0.01), which was further prominently increased with the downregulation of ANGPTL2 (*p* < 0.001) (Figure 4B). Furthermore, the autophagic flux was investigated by transfecting mRFP‐GFP‐LC3 adenovirus into MH‐S cells. The results showed that autophagosomes (labelled with yellow puncta) as well as autolysosomes (appeared as red puncta) both enhanced after MH‐S cells were stimulated with LPS, which both were further markedly increased with the treatment of si ANGPTL2 (*p* < 0.05) (Figure 4C). Thus, downregulation of ANGPTL2 promoted autophagy in LPS‐induced MH‐S cells.

### 3.5 Knockdown of ANGPTL2 attenuated LILRB2‐mediated TREM2 inhibition

**FIGURE 5:**
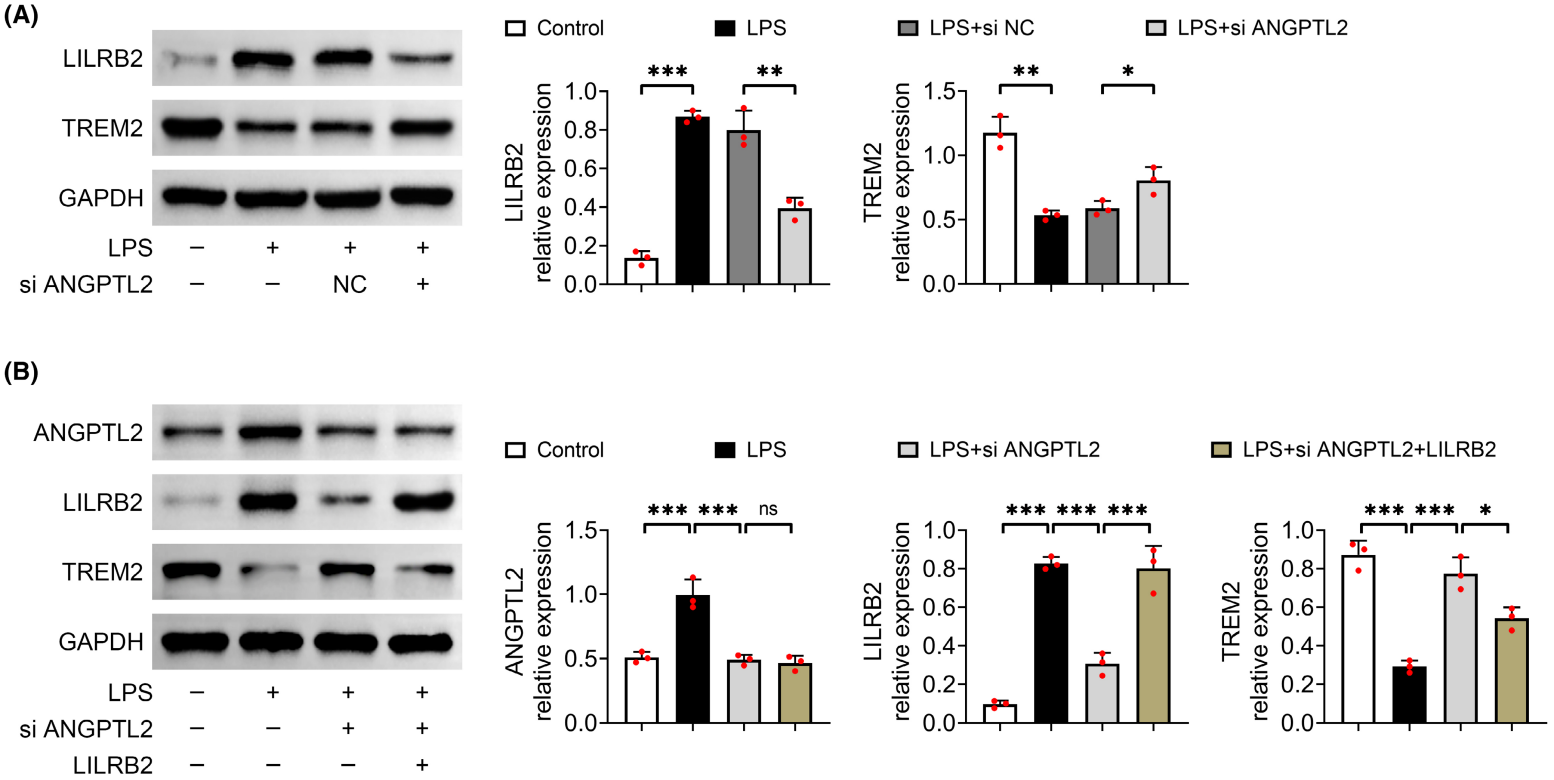
Downregulation of ANGPTL2 reduced LILRB2‐mediated TREM2 inhibition in LPS‐induced MH‐S cells. MH‐S cells were administrated with 10 μg/mL LPS for 4 h to induce lung injury in vitro, and si ANGPTL2 was transfected into MH‐S cells to downregulate the level of ANGPTL2. The sequences of LILRB2 were sub‐cloned into pcDNA vector plasmids for the overexpression of LILRB2. Cells were harvested after 48 h of transfection for the analysis. (A and B) The relative protein expression of ANGPTL2, LILRB2 and TREM2 was examined by western blot. Results were expressed after normalized with GAPDH. Data were shown as averages ± SD. *p < 0.05, **p < 0.01 and ***p < 0.001.

LPS treatment also elicited a remarkable elevation in the relative protein level of LILRB2 (*p* < 0.001), the receptor of ANGPTL2, which was markedly declined by the downregulation of ANGPTL2 (*p* < 0.01) (Figure 5A). Besides, the relative protein expression of TREM2, a pattern recognition receptor widely expressed on macrophages, was prominently reduced in LPS‐induced MH‐S cells (*p* < 0.01), which was notably restored with the knockdown of ANGPTL2 (*p* < 0.05) (Figure 5B). Importantly, overexpression of LILRB2 markedly reduced the relative protein expression of TREM2 after LPS‐induced MH‐S cells were transfected with si ANGPTL2 (*p* < 0.05), but had no statistical influence on the relative protein level of ANGPTL2 (*p* > 0.05) (Figure 5B). Therefore, silencing of ANGPTL2 dampened LILRB2‐mediated TREM2 inhibition in LPS‐induced MH‐S cells.

### 3.6 Downregulation of ANGPTL2 alleviates pyroptosis by upregulating TREM2‐mediated autophagy in LPS‐induced MH‐S cells

**FIGURE 6:**
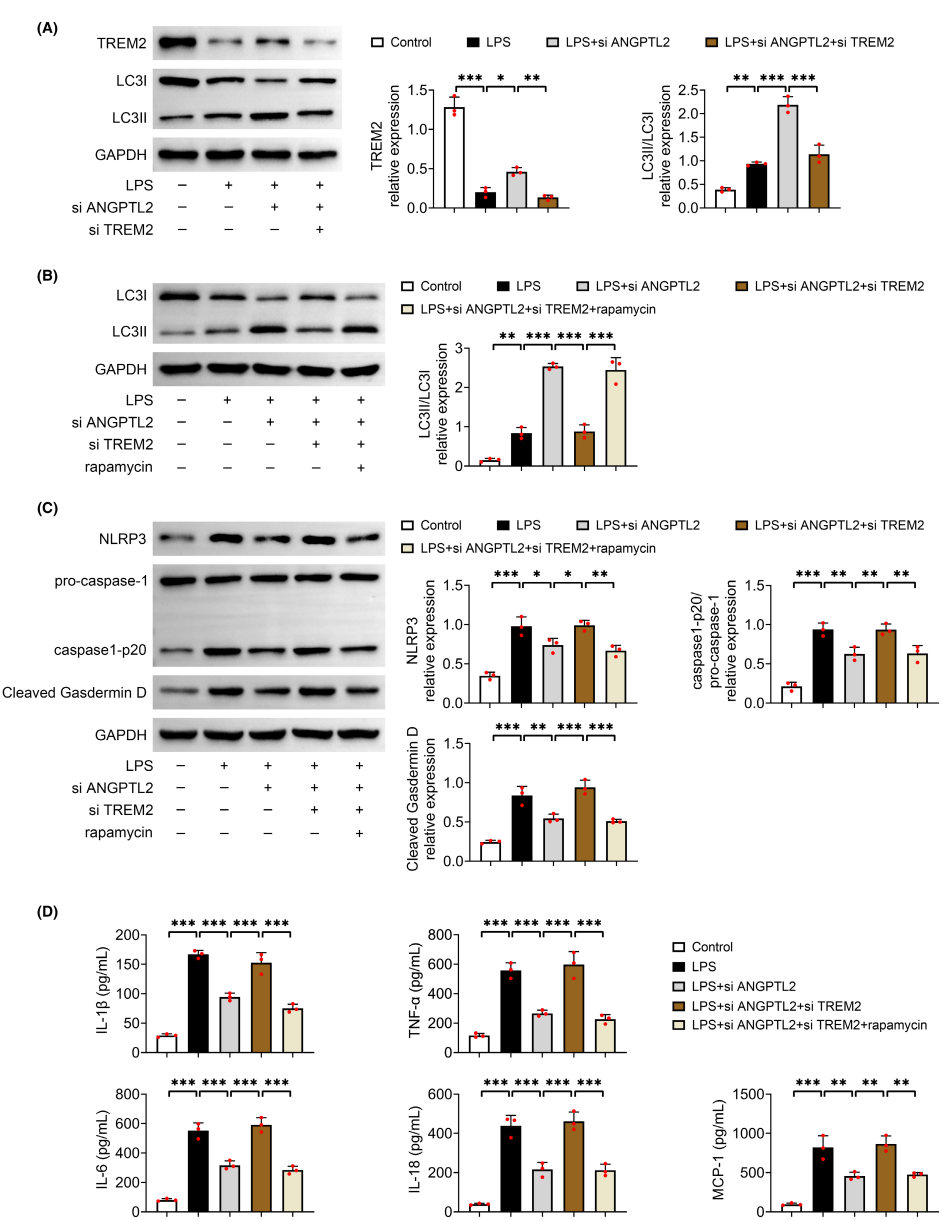
Downregulation of ANGPTL2 mitigated pyroptosis via upregulating TREM2‐mediated autophagy in LPS‐induced MH‐S cells. MH‐S cells were administrated with 10 μg/mL LPS for 4 h to induce lung injury in vitro, and si ANGPTL2 and si TREM2 were transfected into MH‐S cells by using Lipofectamine 3000 to downregulate the level of ANGPTL2 and TREM2, respectively. MH‐S cells were incubated with rapamycin with a concentration of 3 μM for 12 h. (A) The relative protein expression of TREM2, LC3II and LC3I was examined by western blot. Results were expressed after normalized with GAPDH. (B) The relative protein expression of LC3II and LC3I was examined by western blot. Results were expressed after normalized with GAPDH. (C) The relative protein expression of NLRP3, pro‐caspase‐1, caspase1‐p20 and cleaved Gasdermin D in MH‐S cells was determined by western blot. Results were expressed after normalized with GAPDH. (D) The contents of IL‐1β, TNF‐α, IL‐6, IL‐18 and MCP‐1 in cellular supernatant of MH‐S cells were measured by ELISA. Data were shown as averages ± SD. *p < 0.05, **p < 0.01 and ***p < 0.001.

To further inspect the role of ANGPTL2/LILRB2/TREM2 axis in lung injury, the level of TREM2 was downregulated by transfecting si TREM2 into LPS‐induced MH‐S cells after si ANGPTL2 transfection (*p* < 0.01) (Figure 6A). Silencing of ANGPTL2 prominently elevated the relative protein expression of LC3II/LC3I in LPS‐evoked MH‐S cells (*p* < 0.001), which was memorably neutralized with knockdown of TREM2 (*p* < 0.001) (Figure 6A). Moreover, this reduction resulted from knockdown of TREM2 in LPS‐evoked MH‐S cells infected with si ANGPTL2 was markedly recovered by rapamycin, an autophagy activator (*p* < 0.001) (Figure 6B). Thus, knockdown of ANGPTL2 enhanced autophagy by TREM2. Besides, downregulation of ANGPTL2 caused a conspicuous decrease in the relative protein level of NLRP3, pro‐caspase‐1/caspase1‐p20 and cleaved Gasdermin D in LPS‐evoked MH‐S cells (*p* < 0.05), which was markedly rescued by the knockdown of TREM2 (*p* < 0.05) (Figure 6C). However, these rescues were again significantly counteracted with the treatment of rapamycin (*p* < 0.01) (Figure 6C). Parallel results were also shown in the contents of IL‐1β, TNF‐α, IL‐6, IL‐18 and MCP‐1 in cellular supernatant (*p* < 0.01) (Figure 6D). Taken together, silencing of ANGPTL2 relieved pyroptosis by upregulating TREM2‐mediated autophagy in LPS‐evoked MH‐S cells.

## 4 DISCUSSION

ALI/ARDS is characterized with the severe inflammatory response, thus, targeting to inflammation is a significant strategy for the treatment. ANGPTL2, a pro‐inflammatory factor, is notably upregulated in a variety of inflammatory diseases and have been identified as a clinical marker beyond systemic inflammation.^18^ LPS is the most common agents to induce inflammatory response.^35^ LPS has been generally used to develop a clinically correlative model of ALI.^36^, ^37^ Here, the model of ALI was also built on mice and MH‐S cells by the management of LPS in vivo and in vitro, respectively. This study revealed that the level of ANGPTL2 was upregulated in lung injury both in vivo and in vitro. Knockout of ANGPTL2 alleviated LPS‐induced lung injury, and inhibited the inflammatory response by modulating polarization of alveolar macrophages in mice. On the other hand, knockdown of ANGPTL2 reduced pyroptosis, but enhanced autophagy in LPS‐induced MH‐S cells. Besides, downregulation of ANGPTL2 reversed the LPS‐induced the expression of LILRB2 and TREM2, which was reversed by the overexpression of LILRB2 in MH‐S cells. Importantly, knockdown of TREM2 reversed the levels of autophagy‐ and pyroptosis‐involved proteins, and the contents of pro‐inflammatory mediators in LPS‐induced MH‐S cells transfected with si ANGPTL2, which was further inverted with the treatment of rapamycin. Collectively, ANGPTL2 knockdown induces autophagy to relieve alveolar macrophage pyroptosis by reducing LILRB2‐mediated inhibition of TREM2.

Inflammation acts as a momentous role in ALI. Enhanced generation and secretion of inflammatory mediator and excess neutrophil migration are crucial cytopathological features of ALI.^38^ Suppressing the pro‐inflammatory factors release and regulating the immune system can contribute to the amelioration of endothelial function in ALI.^39^, ^40^ Imbalance of inflammation aggravates endothelial or epithelial damage, which causes protein‐rich oedema fluid accessing the alveoli.^41^, ^42^ Histologically, with an exception for severe acute inflammatory response, abundant apoptosis of alveolar epithelial cells is also a characteristic of ALI.^43^ In the current study, LPS treatment caused an elevation in the levels of pro‐inflammatory mediators, containing TNF‐α, IL‐6, IL‐18 and MCP‐1 in BALF, and the numbers of total cells and neutrophils in BALF. LPS injection also induced an increase in the pulmonary W/D weight ratio, indicating than the enhanced oedema was occurred in ALI mice. Besides, the apoptosis rate of alveolar epithelial cells was elevated in lung tissues from LPS‐evoked mice. These outcomes suggested that LPS evoked a mice lung injury model. However, the alterations of all of these indicators caused by LPS treatment were improved with the knockout of ANGPTL2 in mice, along with the previous results reported by Yang et al.^19^ Thus, knockout of ANGPTL2 alleviated lung injury in LPS‐induced mice. More importantly, substantial macrophages infiltration was discovered in LPS‐injected mice, while the ablation of ANGPTL2 visibly decreased the M1 macrophages infiltration into lung tissue. Macrophages act as a vital role in inflammatory processes from initiation to resolution.^11^ Two different forms of macrophages exist, containing M1 and M2 macrophages. M1 macrophages sustain and enhance inflammatory reactions to phagocytose dead cells and bacteria, and recruit other inflammatory cells, while M2 macrophages limit the immune response and setout wound healing.^44^ Therefore, knockout of ANGPTL2 modulated polarization of alveolar macrophages to suppress the pro‐inflammatory cytokines release. Taken together, ANGPTL2 deficiency alleviated lung injury through repressing the pro‐inflammatory factors release in ALI, which was involved in the modulation of the polarization of alveolar macrophages.

In certain conditions, macrophages may experience pyroptosis accompanied with an intense inflammatory reaction. Canonical pyroptosis is a cell death in a caspase‐1‐dependent fashion with some features of apoptosis and necrosis.^45^ Inflammasome that comprises a sensor protein, an adaptor protein and pro‐caspase‐1 is formed during pyroptosis, including NLRP1, NLRP3, NLRC4 and AIM2 inflammasomes.^46^, ^47^ Upon the activation of inflammasome, pro‐caspase‐1 is cleaved to produce caspase‐1 with the activity, resulting in the cleavage of pro‐IL‐1β and pro‐IL‐18 to the mature and secreted forms IL‐1β and IL‐18, severally. Alveolar macrophage pyroptosis has been reported to occur in LPS‐induce ALI mice model.^48^, ^49^, ^50^ Similarly, a conspicuous enhancement in the relative protein level of NLRP3, pro‐caspase‐1/caspase1‐p20 and cleaved Gasdermin D, and the contents of IL‐1β and IL‐18 in the LPS‐treated MH‐S cells, which indicated that LPS evoked canonical pyroptosis in LPS‐treated ALI in vitro. Moreover, LPS evoked an elevation in the concentration of pro‐inflammatory mediators, like TNF‐α and IL‐6. However, all of these promotions were attenuated by knockdown of ANGPTL2 in LPS‐treated MH‐S cells. Collectively, ANGPTL2 deficiency inhibited the pro‐inflammatory cytokines release via suppressing pyroptosis in ALI.

Autophagy is an evolutionarily conserved cellular process that influences innate and adaptive immunity in nearly all human cells, thereby controlling the inflammatory reactions in various disorders.^51^, ^52^ It is revealed that activation of autophagy can prevent LPS‐induced ALI.^53^ Here, LPS treatment induced an increase in the level of cytochrome c and LC3II/LC3I, as well as the autophagic flux. Under the action of Atg4, LC3 precursor is processed into soluble LC3‐I, which is connected to phosphatidylethanolamine (PE) under the action of Atg7 and Atg3 to become lipid‐soluble LC3‐II‐PE, associated with the elongation of the autophagolysosome membrane until autophagolysosome formation.^54^ Thus, the results indicated that an enhanced autophagy occurred in LPS‐induced ALI. However, knockdown of ANGPTL2 further promoted the expression of LC3II/LC3I and the autophagic flux, which suggested that downregulation of ANGPTL2 further increased autophagy in LPS‐induced ALI. The regulative role of ANGPTL2 in autophagy has been reported in diabetic nephropathy, in which knockdown of ANGPTL2 increases the expression of LC3II and beclin1.^55^ Totally, silencing of ANGPTL2 promoted autophagy in LPS‐evoked ALI.

Furthermore, LPS treatment elicited the increase in the expression of LILRB2, which was offset by the knockdown of ANGPTL2. LILRB2 is revealed to be a receptor of ANGPTL2 that can be bound and activated by ANGPTL2.^20^ LILRB2 is tightly related to the progression of different inflammatory diseases.^56^, ^57^, ^58^ Especially, ANGPTL2 has been demonstrated to elicit synovial inflammation via LILRB2.^21^ Moreover, LILRB2 negatively modulates TREM2 signalling.^22^ TREM2 is a signal hub that participates in immune response, which is broadly expressed on the surface of macrophages, dendritic cells, and microglia.^23^ Here, downregulation of ANGPTL2 restored the LPS‐induced the expression of TREM2, and overexpression of LILRB2 reduced the relative protein expression of TREM2 after LPS‐induced MH‐S cells were infected with si ANGPTL2. Thus, silencing of ANGPTL2 dampened LILRB2‐mediated TREM2 inhibition in LPS‐induced ALI. Furthermore, knockdown of TREM2 reversed the levels of autophagy‐ and pyroptosis‐involved proteins, and the contents of pro‐inflammatory mediators in LPS‐induced MH‐S cells transfected with si ANGPTL2, which was further inverted with the treatment of rapamycin. A crosstalk has been shown between pyroptosis and autophagy, in which NLRP3 activation involves in the mutual modulation between pyroptosis and autophagy.^59^ Liu et al^60^ reports that buformin, a hypoglycemic agent, suppresses NLRP3‐mediated pyroptosis via enhancing autophagy in sepsis‐induced ALI. Therefore, these results indicated that ANGPTL2 knockdown induced autophagy to relieve alveolar macrophage pyroptosis by reducing LILRB2‐mediated inhibition of TREM2.

In summary, the outcomes of the present study offer the significant evidence that ANGPTL2 deficiency alleviated lung injury. At the molecular level, downregulation of ANGPTL2 attenuated apoptosis, inflammation and pyroptosis, but induced autophagy in LPS‐induce ALI. Mechanically, silencing of ANGPTL2 dampened LILRB2‐mediated TREM2 inhibition in LPS‐induced ALI. However, several limitations remain to be further addressed in the future. Firstly, the clinical role of ANGPTL2 can be investigated in the following study through collecting the clinical data and samples from patients with ALI. Additionally, the detailed role of ANGPTL2 in the polarization of alveolar macrophages should be explored in the subsequent assays. Moreover, more pre‐clinical and clinical experiments are essential to solidify our results. In briefly, our findings shed more probabilities on the treatment of ALI in clinical practice.

## AUTHOR CONTRIBUTIONS

**Fan Yang:** Data curation (equal). **Muhu Chen:** Formal analysis (equal). **Ying Liu:** Formal analysis (equal). **Yingchun Hu:** Formal analysis (equal). **Yangxi Chen:** Methodology (equal). **Youwei Yu:** Investigation (equal). **Lu Deng:** Conceptualization (equal).

## FUNDING INFORMATION

This work was supported by the Sichuan province science and technology plan project. (Grant No. 2022YFS0276).

## CONFLICT OF INTEREST STATEMENT

The authors state that there are no conflicts of interest to disclose.

## Data Availability

All data generated or analysed during this study are included in this published article. The datasets used and/or analysed during the present study are available from the corresponding author on reasonable request.
